# Tumor-Specific Zr-89 Immuno-PET Imaging in a Human Bladder Cancer Model

**DOI:** 10.1007/s11307-018-1177-z

**Published:** 2018-03-05

**Authors:** Freddy E. Escorcia, Jeffrey M. Steckler, Dalya Abdel-Atti, Eric W. Price, Sean D. Carlin, Wolfgang W. Scholz, Jason S. Lewis, Jacob L. Houghton

**Affiliations:** 1 Department of Radiology, Memorial Sloan Kettering Cancer Center, 1275 York Avenue, New York, NY 10065 USA; 2 Department of Radiation Oncology, Memorial Sloan Kettering Cancer Center, 1275 York Avenue, New York, NY 10065 USA; 3 Weill Cornell Medical College, 1300 York Ave, New York, NY 10065 USA; 4 Department of Chemistry, University of Saskatchewan, Saskatoon, SK Canada; 5 MabVax Therapeutics, 11535 Sorrento Valley Rd, San Diego, California, 92121 USA; 6 Program in Molecular Pharmacology, Memorial Sloan Kettering Cancer Center, 1275 York Avenue, New York, NY 10065 USA; 7 Department of Radiology and Radiological Sciences, Vanderbilt University Medical Center, Nashville, TN 37232 USA

**Keywords:** Immuno-PET, Bladder cancer, Molecular imaging, Antibody, Zirconium-89

## Abstract

**Purpose:**

Tumor-specific molecular imaging is an important tool for assessing disease burden and treatment response. CA19.9 is an important tumor-specific marker in several malignancies, including urothelial carcinoma. [^89^Zr]DFO-HuMab-5B1 (MVT-2163) is a CA19.9-specific antibody-based construct that has been validated in preclinical animal models of lung, colorectal, and pancreatic malignancies for positron emission tomography (PET) imaging and is currently in a phase I trial for pancreatic cancer (NCT02687230). Here, we examine whether [^89^Zr]DFO-HuMab-5B1 may be useful in defining urothelial malignancies.

**Procedures:**

Surface expression of CA19.9 was confirmed in the human bladder cancer line HT 1197. The radioimmunoconjugate [^89^Zr]DFO-HuMab-5B1 was injected into mice bearing HT 1197 xenografts, and followed by PET imaging, *ex vivo* experiments including biodistribution, histology and autoradiography, and analysis of blood samples for shed antigen levels were performed.

**Results:**

[^89^Zr]DFO-HuMab-5B1 specifically accumulates in HT 1197 engrafted tumors when imaged with PET. *Ex vivo* biodistribution of organs and autoradiography of engrafted tumors confirm our construct’s specific tumor binding. The target antigen CA19.9 was not found to be shed *in vitro* or *in vivo*.

**Conclusions:**

[^89^Zr]DFO-HuMab-5B1 can be used to delineate urothelial carcinomas by PET imaging and may provide tumor-specific information prior to, during, and after systemic therapies.

## Introduction

This year, there will be an estimated 79,030 new cases of and 16,870 deaths from urothelial carcinoma in the USA [1]. While the majority of patients with bladder cancers present in early stages, those with locally advanced or metastatic disease have a 5-year survival rate of only 5 % [2]. First-line therapy for these patients is combination chemotherapy with methotrexate, vinblastine, doxorubicin, and cisplatin (MVAC) or doublet therapy with gemcitabine plus cisplatin. These treatments have been shown to result in both higher overall response rates as well as improved overall survival [3]. In addition to standard cytotoxic therapy, the recent approval of antibodies targeting an immune checkpoint for patients with metastatic disease reaffirms the importance of host immunity in bladder cancer, especially since therapy for early-stage disease has long included an immune modulator, Bacillus Calmette-Guerín (BCG) [4]. Furthermore, novel therapeutic interventions targeting key oncogenic pathways specifically altered in urothelial carcinoma expanded our therapeutic armamentarium and allow for precision tailoring of treatments for individual patients [5, 6].

Taking tumor-specific features and devising therapeutic interventions that exploit differences between cancer and normal tissues optimizes the therapeutic ratio and is the ultimate goal of any treatment. This approach is also paramount for engineering tumor-specific molecular imaging agents, which can (1) help define micrometastatic disease, (2) provide additional important biological information about the tumor beyond glucose uptake (*e.g.*, standard 2-deoxy-2-[^18^F]Fluoro-d-glucose ([^18^F]FDG) PET), and (3) allow for monitoring of treatment response. Additional value can be derived from successful molecular imaging agents, such as PET radioimmunoconjugates, because once proven successful for immno-PET imaging, they can be modified for radiolabeling with radiotherapeutic isotopes such as Lu-177, Y-90, Pb-212, or Ac-225.

Carbohydrate antigen 19.9 (CA 19.9) is a common tumor-associated antigen of hepatobiliary, colorectal, lung, and bladder cancers [7]. Serum CA19.9 is significantly increased in 35–58 % of patients with known urothelial carcinoma and is significantly increased in > 70 % of muscle-invasive cases *versus* non-muscle-invasive cancers, making a potentially important prognostic marker [2, 7–9]. While several serum and cytology markers for urothethial cancers have been identified, they lack the sensitivity needed for appropriate screening [10]. In fact, serum CA 19.9 suffers similar limitations. To address these limitations, we have previously demonstrated the value in being able to image this antigen *in situ.* Our group has taken HuMab-5B1 (MVT-5873), a CA19.9-specific human antibody, and confirmed the feasibility and efficacy of targeting CA19.9 for both imaging and therapy in pancreatic cancer models [7, 11, 12]. Here, we demonstrate the utility of our immuno-PET probe, [^89^Zr]DFO-HuMab-5B1 (MVT-2163), which, like serum CA19.9, can confirm the presence of CA19.9-expressing human bladder cancer cells, but, unlike serum CA19.9, also identifies the specific anatomical location of these lesions *in situ*, *in vivo*, and in real time, and unlike [^18^F]FDG PET, is tumor specific.

## Methods

### Cell Culture

HT 1197 and BxPC3 cells (ATCC, Manassas, VA) were grown according to standard methods described by supplier. All media were purchased from the Media Preparation Facility at Memorial Sloan Kettering Cancer Center.

### Flow Cytometry

Approximately 10 × 10^6^ HT 1197 cells were harvested and washed with ice-cold PBS three times. Cell pellet was resuspended in FcR block (Miltenyi Biotec, Bergisch Gladbach, Germany) and incubated for 30 min on ice. Cell suspension was then split into multiple groups, stained with fluorescently labeled HuMab-5B1 (3 μg/ml), stained with fluorescently labeled IgG-isotype control (3 μg/ml), or incubated without antibody for 30 min on ice. Following incubation, cells were washed with ice-cold FACS buffer (phosphate-buffered saline, 2 % FCS, 0.1 % sodium azide, 1 mM ethylenediaminetetraacetic acid (EDTA)) three times. DAPI (Sigma-Aldrich, St. Louis, MO) was added prior to assaying samples. Single-color controls were made and results were analyzed with FlowJo software (FlowJo LLC, Ashland, Oregon).

### Immunofluorescence Staining

HT 1197 cells were plated on 8-well chamber slides at a density of 2 × 10^5^ per well and incubated overnight. Chambers were washed with PBS three times. Cells were fixed with 4 % paraformaldehyde for 10 min on ice, then washed with PBS three times, and incubated with blocking buffer [1 % bovine serum albumin in phosphate-buffered saline (PBS)] for 5 min. After three washes with PBS, slides were incubated with fluorescently labeled HuMab-5B1 antibody for 20 min at 40 °C. Following incubation, cells were washed five times with PBS, counterstained with DAPI for 15 min, and washed with dH_2_O. Gasket and rubber seal were removed and slide was rinsed with dH_2_O. Samples were covered with Mowiol mounting media followed by a coverslip, and the slides were stored at − 20 °C prior to imaging.

### Radiolabeling of HuMab-5B1 with Zr-89

HuMab-5B1 was produced and provided by MabVax Therapeutics (San Diego, CA) Preparation of Zr-89-labeled HuMab-5B1 ([^89^Zr]DFO-HuMab-5B1 or MVT-2163) was achieved in accordance with previously described methods, including conjugation of *p*-SCN-Bn-DFO, purification, and subsequent radiolabeling [13]. Briefly, Zr-89 oxalate in oxalic acid (1 M) was neutralized to pH 7.0–7.2, using Na_2_CO_3_ (1 M) followed by addition of the appropriate construct in PBS (pH 7.4). The mixture was incubated at room temperature (RT) for 30–60 min and monitored using radio-iTLC with silica-gel impregnated glass-microfiber paper strips (iTLC-SG, Varian, Lake Forest, CA, analyzed using an AR-2000, Bioscan Inc., Washington, DC), eluted with a mobile phase of aqueous solution of EDTA (50 mM, pH 5.5). The reaction was quenched by addition of EDTA solution (10–20 μl). Gel-filtration chromatography (Sephadex G-25, PD10 desalting column; GE Healthcare, Chicago, IL) with 0.9 % saline was used to purify radiolabeled construct, and radiochemical purity was determined by radio-iTLC as described above. Zr-89 was obtained from and produced by MSKCC *via* the ^89^Y(*p*,*n*)^89^Zr transmutation reaction using a TR19/9 variable-beam energy cyclotron (Ebco Industries, Richmond, British Columbia, Canada) [14].

### Immunoreactivity Measurements

The immunoreactivity of the Zr-89-labeled construct, [^89^Zr]DFO-HuMab-5B1, was determined using BxPC3, a human pancreatic cancer cell line which highly expresses CA19.9, *via* a previously reported method [15, 16]. Data were background-corrected and the ratio of the total to bound (total/bound) radioactivity was plotted against the inverse of the normalized cell concentration (1/normalized cell concentration) for the linear regression analysis.

### Murine Subcutaneous Xenograft Models

Female athymic homozygous nude mice, strain Crl:NU(NCr)-Foxn1^nu^ (Charles River Laboratories, Wilmington, MA), age between 6 and 8 weeks, were xenografted subcutaneously with 5 × 10^6^ HT 1197 cells, suspended in 150 μl of a solution containing a 1:1 mixture of Matrigel (Corning, Corning, NY) and cell culture medium. Tumors were grown to a size of approximately 150 mm^3^ post-implantation before imaging.

### PET Imaging

For experiments with the HT 1197 subcutaneous xenograft model, mice (*n* = 3) were administered [^89^Zr]DFO-HuMab-5B1 [3.0–4.0 megabecquerels (MBq) (50–110 μCi/10–20 μg)] *via* tail vein injection. In the case of blocking studies, 25-fold mass excess of HuMab-5B1 was administered 48 h prior to injection with [^89^Zr]DFO-HuMab-5B1. A microPET Focus 120 scanner (Concorde Microsystems, Knoxville, Tennessee) was used to obtain static scans at the desired time points with at least 5–10 million coincident events and ASIPro VM software was used to analyze acquired images.

### Biodistribution

The acute biodistribution of [^89^Zr]DFO-HuMab-5B1 was determined using the same HT 1197 subcutaneous (right flank, 150 mm^3^) model as used for PET imaging (athymic, nude mice). Mice tumor volumes were measured prior to imaging using a Peira TM900 tumor-measuring device, and the mice were separated into groups with similar mean tumor volumes before being injected *via* the lateral tail vein (52.3 ± 1.0 μCi/10 μg). At 48 and 120 h post-injection, mice (*n* = 5) were euthanized. Thirteen tissues including the tumor were collected. Each sample was weighed and a Wizard^2^ automatic gamma counter calibrated for Zr-89 was used to measure the corresponding radioactivity. The counts from each sample were decay- and background-corrected, and counts were converted into activity by using a calibration curve generated from Zr-89 standards of known activity. The % ID/g was calculated by normalizing data to the total activity injected into the corresponding animal.

### Autoradiography and Histology

Tumors of euthanized animals were excised, submerged in Tissue–Plus OCT compound (Scigen, Gardena, CA), and frozen on dry ice. Series of 10 μm tissue sections were cut and placed in a film cassette beneath a phosphor-imaging plate for 48–72 h at − 20 °C (BASMS-2325; Fujifilm, Tokyo, Japan), which was then read on a Typhoon 7000IP plate reader (GE Healthcare, Chicago, IL) at 25 μm pixel resolution. Several sequential sections were submitted to the Molecular Cytology Core Facility at MSKCC for hematoxylin and eosin and HuMab-5B1 (0.5 μg/ml) staining. Image analysis was performed with ImageJ (https://imagej.nih.gov/ij/).

### Microscopy

A Zeiss Axioplan2 fluorescence microscope connected to a CCD camera and equipped with a motorized stage (Prior Scientific Instruments, Rockland, MA) was used to acquire microscopic images. MetaMorph software (Molecular Devices, Sunnyvale, CA) was used to control the microscope as well as to generate a montage of the entire tumor from the captured image frames. Photoshop CS6 software (Adobe Systems, McLean, VA) was used for post-acquisition processing.

### Assessing Shedding Status of CA19.9

Whole blood was collected from mice xenografted with HT 1197 cells at several time points post tumor cell inoculation (*n* = 4 per time point) *via* retro-orbital bleeds with capillary tubes. Blood was transferred immediately to 1.5 ml microcentrifuge tubes and allowed to clot for 1 h at RT. Gross clot was removed and remaining sample spun down at 1000×*g* × 10 min. Supernatant was collected and stored at − 20 °C until ELISA (Affymetrix no. BC1017, Santa Clara, CA) was performed per manufacturer protocol. Positive controls of predetermined CA19.9 concentration and media from T175 flasks containing confluent HT 1197 were also assayed.

### Statistical Analysis

Quantitation of results was performed with Prism software (Version 7.0, GraphPad software, La Jolla, CA). An unpaired, two-tailed Student’s *t* test was used to analyze the data. In all cases, a 95 % confidence level (*P* < 0.05) was considered to represent a statistical difference in the data.

## Results

### HuMab-5B1 Binds to Human Bladder Cancer Line

**Fig. 1:**
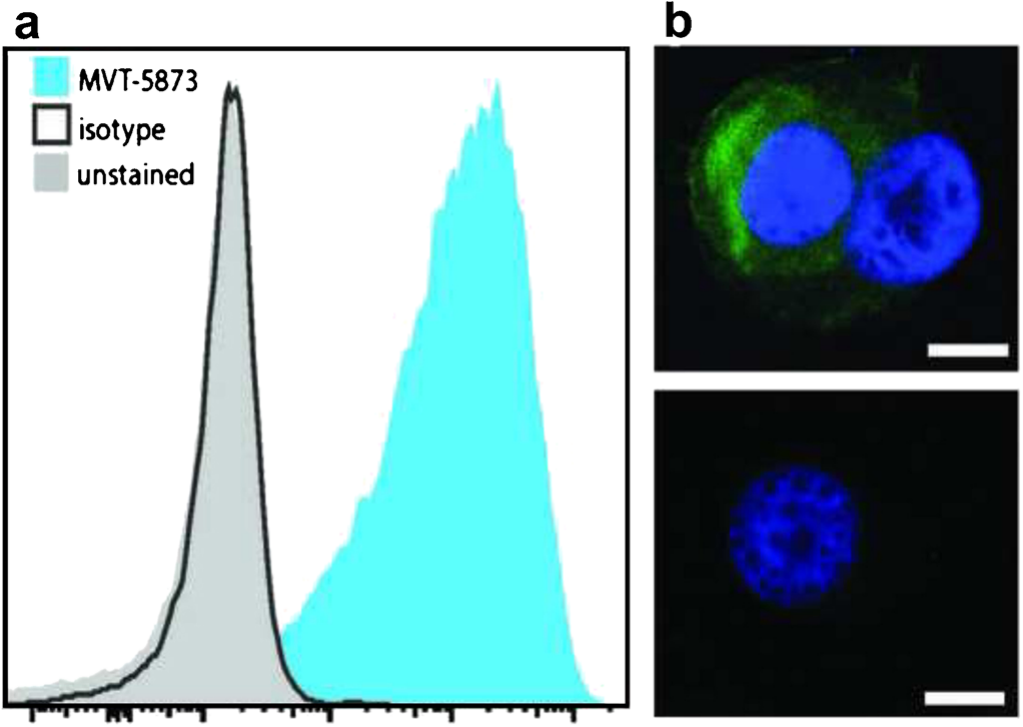
HuMab-5B1 binds to CA19.9-expressing HT 1197 cells. **a** Flow cytometry histogram of HT 1197 cells incubated with HuMab-5B1 labeled with AlexaFluor488 (AF488) dye (blue), isotype IgG labeled with AF488 (black line), or without antibodies (light gray). **b** Immunofluorescence images of HT 1197 cells stained with HuMab-5B1-IR650 (top) or isotype IgG-IR650 (bottom). Scale bar represents 10 μm.

To confirm presence of CA19.9 on human bladder cancer, flow cytometry and immunofluorescent studies of cells incubated with fluorescently labeled HuMab-5B1 were performed. Flow cytometry histogram (Fig. 1a) and immunofluorescence imaging (Fig. 1b) demonstrate abundant expression of CA19.9 on HT 1197 cell lines as evidenced by binding of fluorescently labeled HuMab-5B1. In contrast, when cells are incubated with fluorescently labeled isotype control IgG, no significant binding is observed (Fig. 1).

#### [^89^Zr]DFO-HuMab-5B1 Localizes to CA19.9-Expressing HT 1197 Tumors *In Vivo*

**Fig. 2:**
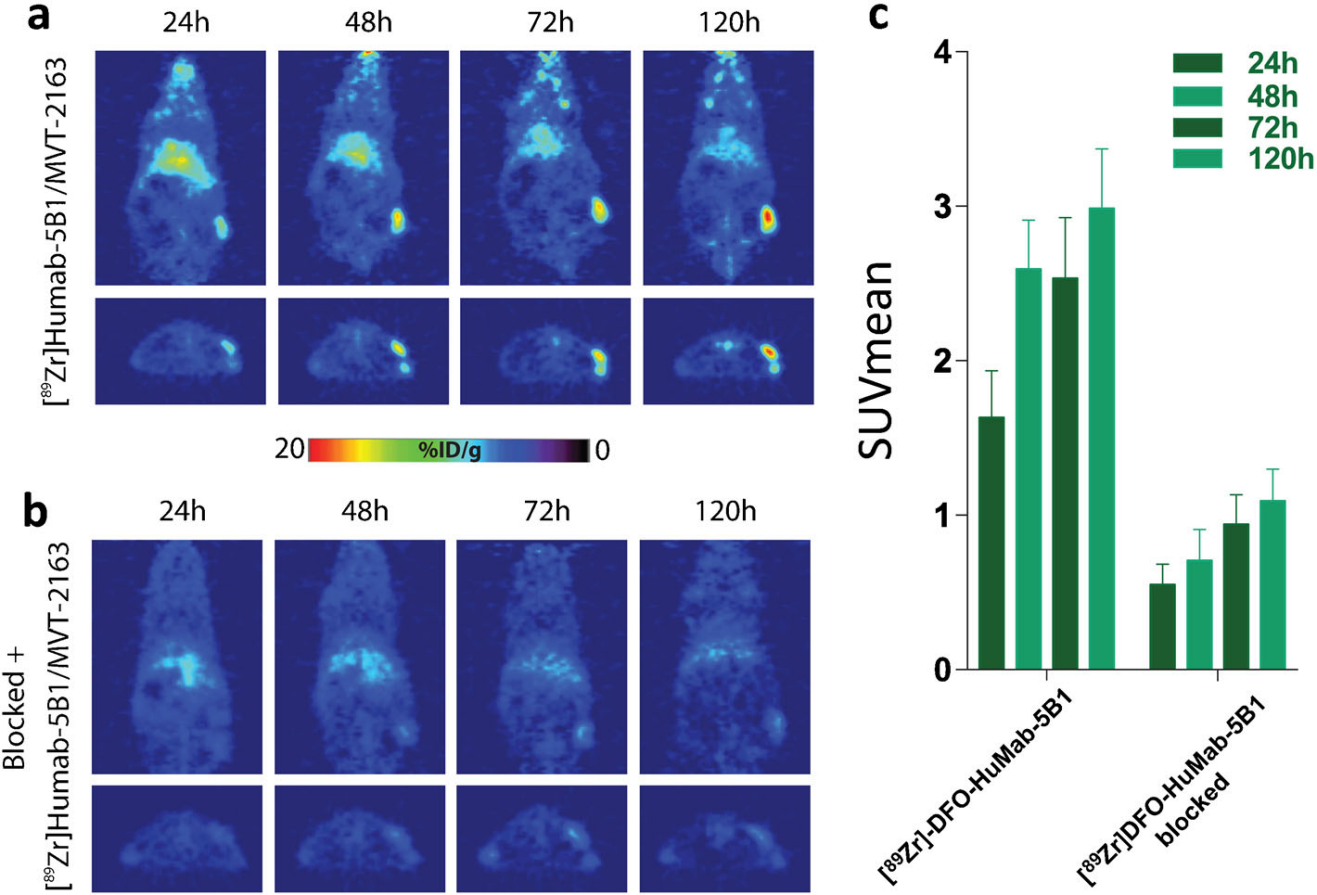
[^89^Zr]DFO-HuMab-5B1 specifically localizes to CA19.9-expressing human bladder cancer. **a** Representative images from animals with subcutaneous HT 1197 xenografts at varying intervals post-injection with [^89^Zr]DFO-HuMab-5B1 radiotracer. **b** Animals pretreated with 25-fold excess unlabeled HuMab-5B1 (MVT-5873) demonstrated only non-specific accumulation of [^89^Zr]DFO-HuMab-5B1 tracer. **c** Region of interest (*n* = 3 per time point) quantitation of PET images confirm higher tumor SUV_mean_ [^89^Zr]DFO-HuMab-5B1 alone *versus* when blocked with HuMab-5B1 (1.6 ± 0.5 *versus* 0.5 ± 0.2 at 24 h, 2.6 ± 0.5 *versus* 0.7 ± 04 at 48 h, 2.5 ± 0.7 *versus* 0.9 ± 0.3 at 72 h, and 3.0 ± 0.7 *versus* 1.01 ± 0.4 at 120 h).

**Fig. 3:**
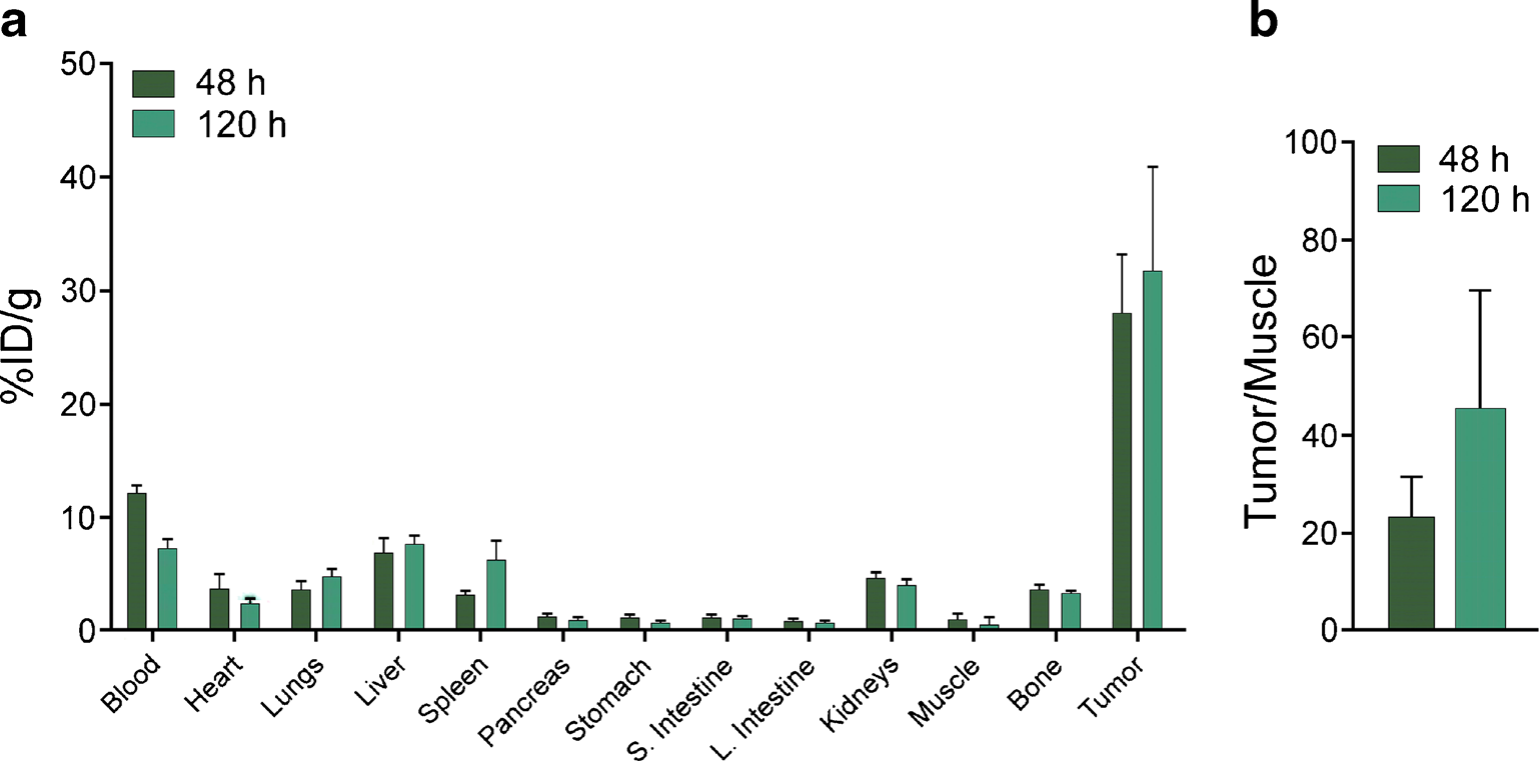
[^89^Zr]DFO-HuMab-5B1 specifically localizes to CA19.9-expressing human bladder cancer with minimal accumulation in non-target tissues. **a** *Ex vivo* biodistribution and **b** tumor-to-muscle ratio at 48 and 120 h following injection with [^89^Zr]DFO-HuMab-5B1 (MVT-2163). *N* = 5 per group. %ID/g = percent injected dose per gram of tissue.

[^89^Zr]DFO- HuMab-5B1 was synthesized with radiochemical purity and yield comparable to previous reports, > 98 and > 90 %, respectively [7, 17]. Immunoreactivity was determined to be > 90 % using the Lindmo assay [15, 16] and was comparable to previously reported values with the same cell lines. PET imaging shows specific accumulation and retention of [^89^Zr]DFO-HuMab-5B1 in HT 1197 tumor-bearing mice over 24 to 120 h post-injection (Fig. 2c). Rapid accumulation in tumors is observed and persists to at least 120 h (Fig. 2a). Quantitation of PET images by analyzing regions of interest (*n* = 3 per time point) demonstrate higher tumor SUV_mean_ in [^89^Zr]DFO-HuMab-5B1-treated group *versus* the blocked group, peaking at 120 h, with values of 3.0 ± 0.7 *versus* 1.01 ± 0.4, respectively (Fig. 2c). Notably, signal observed at bilateral hind joints is consistent with free, unchelated Zr-89. *Ex vivo* biodistribution and quantitation demonstrates average tumor percent-injected dose per gram (%ID/g) values of 28.1 ± 11.3 and 31.8 ± 20.2 at 48 and 120 h post-injection, respectively (Fig. 3a). Tumor/muscle values demonstrate favorable signal-to-background contrast with values of 23.4 ± 18.0 and 45.4 ± 53.9 at 48 and 120 h post-injection, respectively (Fig. 3b).

### Autoradiography and Microscopy Show *In Situ* Target Engagement

**Fig. 4:**
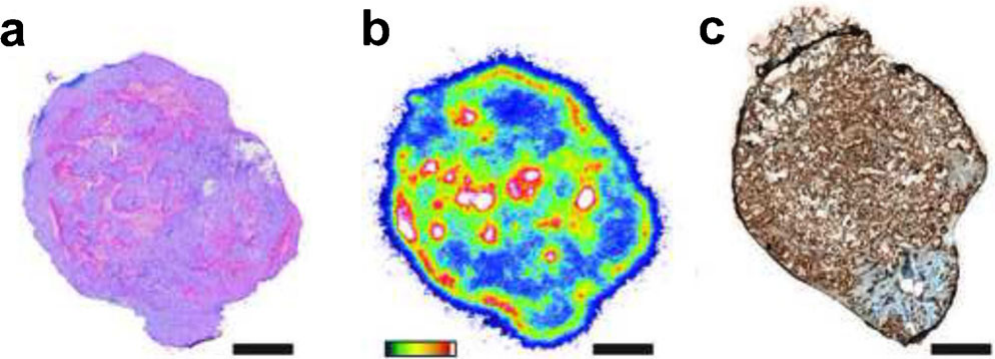
HT1197 tumors demonstrates specific accumulation of [^89^Zr]DFO-HuMab-5B1. **a** Hematoxylin and Eosin-stained tumor. **b** False color autoradiography (high activity white, low activity blue). **c** CA19.9 expression (brown). Adjacent tumor sections were utilized. Scale bar represents 2 mm.

Tumors were harvested and sections were assessed for H&E, autoradiography, and CA19.9 staining. Results demonstrate heterogeneous distribution of activity across the entire tumor section coincident the glandular structures, and diffuse staining for CA19.9 (Fig. 4).

#### Sera of HT 1197-Engrafted Animals Do Not Demonstrate Evidence of Shed Antigen

**Fig. 5:**
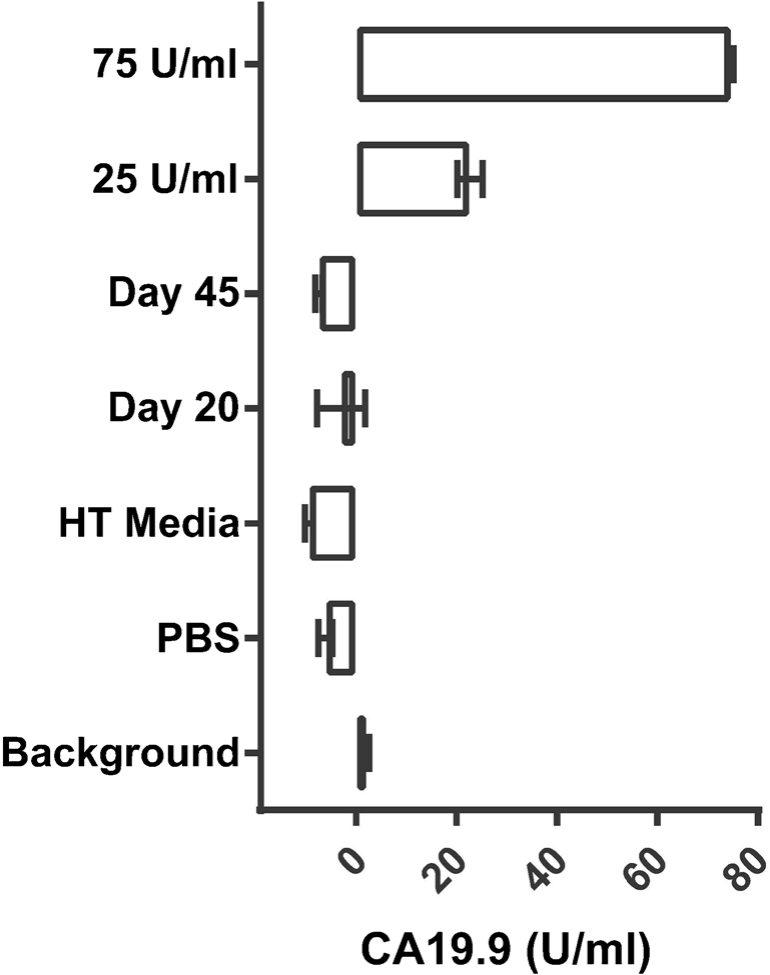
HT 1197 cells do not shed CA19.9. ELISA performed on HT 1197 media, and serum HT 1197 engrafted mice at days 20 and 45 post implantation demonstrates CA19.9 levels comparable to background. Sera of four animals were evaluated per group. Media, PBS, and background were assessed in duplicates. Assay standards for CA19.9 (25 and 75 U/ml) were included for comparison.

To determine whether HT 1197 cells shed CA19.9, serum samples from mice engrafted with HT 1197 cells assessed at days 20 (*n* = 4) and 45 (*n* = 4) after engraftment and of media from confluent flasks containing HT 1197 (HT media) were measured *via* ELISA. When plotted against assay negative (background, PBS) and positive (25 and 75 U/ml) controls, results demonstrated undetectable CA19.9 levels when compared to assay standards, 25 and 75 U/ml, suggesting that HT 1197 cells do not shed CA19.9 neither *in vitro* nor *in vivo* (Fig. 5).

## Discussion

The ability to better delineate tumors based on tumor-specific markers is paramount to clinical decision-making. As we discover new targets (*e.g.*, receptors, enzymes) and deploy methods to generate imaging probes to these molecules, we can contribute to the refining of tumor classification beyond existing paradigms, and, hopefully, help personalize treatment choices accordingly.

Cystoscopy is most useful for early diagnosis of bladder cancer. Approximately 70–80 % of newly diagnosed patients present superficial bladder tumors, and the prognosis of these patients depends largely on the grade of the tumor (https://www.cancer.gov/types/bladder/hp/bladder-treatment-pdq). The majority of deaths from bladder cancer are due to high-grade disease where cancer cells invade the muscular wall of the bladder and spread to other parts of the body. Patients with high-grade disease are typically staged with a CT scan of the chest, abdomen, and pelvis, and patients with suspected bone metastases undergo a bone scan.

While [^18^F]FDG PET is also utilized to identify and monitor local or metastatic tumors of various histological types, including bladder cancer, intravesicular lesions are difficult to assess with this modality because [^18^F]FDG is excreted renally and accumulates in the bladder. As such, [^18^F]FDG PET is more commonly employed in the metastatic setting, exploiting preferential glucose uptake in rapidly dividing cells. It is well known that normal tissues can also take up [^18^F]FDG, especially following surgical procedures, in the setting of inflammation or physiologic rapid cell turnover. Accordingly, developing tumor-selective functional imaging can provide valuable information about tumor aggressiveness and propensity for dissemination, which is typically unavailable with standard imaging.

We have previously reported on the synthesis of our CA19.9-targeting immuno-PET construct, ^89^Zr-DFO-HuMab-5B1, and its ability to specifically target human pancreatic, colorectal, and lung cancers in a xenograft animal model system [7, 11, 12, 18]. Here, we show that HuMab-5B1 binds to human bladder cancer cells *in vitro,* and PET imaging with [^89^Zr]DFO-HuMab-5B1 demonstrates tumor localization *in vivo*. Tumor localization was further confirmed *ex vivo* *via* organ biodistribution and tumor autoradiographic studies. A majority of bladder cancers express CA19.9, and this work provides a new potential PET imaging approach for visualizing invasive bladder cancers expressing high levels of CA19.9 in patients [8]. While prior studies demonstrated utility of imaging bladder cancer-selective agents, to our knowledge, the antigen CA19.9 has not been previously used as a target for PET imaging of this disease [19].

Notably, our studies indicate that, while we are able to get excellent localization in bladder tumors, there is also accumulation in the bones of our experimental animals. This is a well-known, previously described drawback of the Zr-89-DFO complex, and we and others are investigating alternative Zr-89 chelation strategies to mitigate this phenomenon [20–22]. Uptake of [^89^Zr]DFO-HuMab-5B1 in CA19.9 overexpressing murine xenograft models of bladder cancer (HT 1197) was high by PET visualization and by *ex vivo* biodistribution, with peak uptake at 120 h p.i. of 31.8 ± 20.2 %ID/g. Autoradiography and immunohistochemical staining has confirmed co-localization in HT 1197 tumor xenografts of the radioactive [^89^Zr]DFO-HuMab-5B1 construct with high CA19.9 expression.

Most bladder cancers are transitional cell carcinomas arising from transitional epithelium, a specialized mucous membrane. Serum levels of CA19.9 are moderately increased in patients with advanced and metastatic bladder cancer [9, 10]. It is well documented that the CA19.9 antigen in serum is displayed on mucin; however, not all mucins are shed from the cell surface [23, 24]. Notably, heterogeneity of antigen shedding is observed with various cell lines, and we find that the HT 1197 cell line neither sheds CA19.9 in *in vitro* nor *in vivo* [7].

Target shedding or cleavage from tumor, as can occur with several tumor-associated antigens that are subsequently detected with serum assays, can in principle diminish *in situ* tumor localization, degrade signal-to-noise in imaging studies, and diminish the therapeutic ratios when utilizing therapeutic radionuclides. While it is important to assess the shedding status of this antigen in our model system, we understand that individual patient tumor types may differ in CA19.9 shedding. However, herein lies the advantage of the HuMab-5B1 PET imaging agent: HuMab-5B1 is highly internalized and can localize to tumor despite circulating shed antigen [11]. Additionally, the hurdle of targeting the tumor tissues in the presence of shed CA19.9 in the serum is easily overcome by preloading with unmodified 5B1 [17]. Thus, the PET imaging agent can provide information about tumor location and extent of metastasis which cannot be gathered from the serum biomarker alone. Tumor-specific PET imaging could be used for staging and selection of precision treatment regimens of patients. Moreover, fluorescence-guided surgery is gaining increased interest and it may be worthwhile to examine whether fluorescent dye-labeled HuMab-5B1 could be used to guide partial cystectomy [25].

Studies are currently ongoing to assess our construct in orthotopic models of this cell line and assess the contribution of the microenvironment on antigen targeting. Concurrently, [^89^Zr]DFO-HuMab-5B1 is being evaluated in an ongoing clinical trial in patients with pancreatic cancer or other CA19-9-positive malignancies (clinicaltrials.gov, NCT02687230). Our current study confirms that the [^89^Zr]DFO-HuMab-5B1 radioimmunoconjugate can detect human urothelial cancer, adding preclinical rationale for utility in this aggressive cancer type, especially in patients with elevated CA19.9 levels. We believe that this construct can serve as an important diagnostic tool for assessing tumor-specific responses prior to, during, and after systemic therapies and has the potential to guide the development of targeted bladder cancer therapies.
